# MLK3 Signaling in Cancer Invasion

**DOI:** 10.3390/cancers8050051

**Published:** 2016-05-19

**Authors:** Chotirat Rattanasinchai, Kathleen A. Gallo

**Affiliations:** 1 Cell and Molecular Biology program, Michigan State University, East Lansing, MI 48824, USA; rattana1@msu.edu; 2 Department of Physiology, Michigan State University, East Lansing, MI 48824, USA

**Keywords:** cancer invasion, signal transduction, mixed lineage kinase 3 (MLK3), metastasis

## Abstract

Mixed-lineage kinase 3 (MLK3) was first cloned in 1994; however, only in the past decade has MLK3 become recognized as a player in oncogenic signaling. MLK3 is a mitogen-activated protein kinase kinase kinase (MAP3K) that mediates signals from several cell surface receptors including receptor tyrosine kinases (RTKs), chemokine receptors, and cytokine receptors. Once activated, MLK3 transduces signals to multiple downstream pathways, primarily to c-Jun terminal kinase (JNK) MAPK, as well as to extracellular-signal-regulated kinase (ERK) MAPK, P38 MAPK, and NF-κB, resulting in both transcriptional and post-translational regulation of multiple effector proteins. In several types of cancer, MLK3 signaling is implicated in promoting cell proliferation, as well as driving cell migration, invasion and metastasis.

## 1. Introduction

Metastasis is the major cause of death in many cancer types. Despite improvements in cancer therapeutics, which have proven effective in early stage cancer, metastatic disease remains incurable and patients with metastatic cancer have a poorer outcome [1]. The process of metastasis consists of multiple steps and is considered fairly inefficient, as failure to complete any step of this process will prevent metastatic disease [2,3]. Acquisition of genetic and/or epigenetic alterations in primary tumors, which causes a subset of cancer cells to gain invasive capability, is thought to initiate the metastatic process. Only a subset of cancer cells capable of degrading the extracellular matrix (ECM) can disseminate into the ECM surrounding the primary tumor. To form distant metastases, cancer cells must be able to intravasate into blood vessels, survive and circulate within the bloodstream, and extravasate out at a distant site. Only if the new microenvironment is favorable will these cancer cells colonize and form a distant metastatic tumor [4].

Genetic and epigenetic alterations disrupt normal cellular signaling pathways by altering gene expression or protein activity in cancer cells. Dysregulation of the mitogen-activated protein kinase (MAPK) pathway has been reported in several types of cancer and is involved in multiple aspects of cancer progression [5,6]. The MAPK signaling cascade is an evolutionarily conserved pathway and is important for many biological processes such as cell survival, cell proliferation, cell differentiation and cell motility [7,8,9,10]. Members of the MAPK cascade function as signal mediators, transmitting extracellular signals to intracellular substrates. In normal cells, MAPK signaling, triggered upon mitogen stimulation, is tightly controlled by a three-tiered phosphorelay of protein kinases. Once stimulated, MAP kinase kinase kinases (MAP3Ks) phosphorylate and activate specific MAP kinase kinases (MAP2Ks, MKKs), which in turn phosphorylate and activate specific MAP kinases (MAPKs). Depending upon the context and cell type, MAPK proteins can either phosphorylate cytosolic substrates or translocate into the nucleus to phosphorylate and regulate transcription factors. Overexpression and constitutively-active mutated MAPK proteins as well as their upstream regulators are commonly present in various types of cancers. Notably, these alterations lead to upregulation of MAPK pathway and sustained signaling for cell survival, proliferation, migration and invasion of cancer cells.

## 2. The Mixed Lineage Kinase (MLK) Family

**Figure 1:**
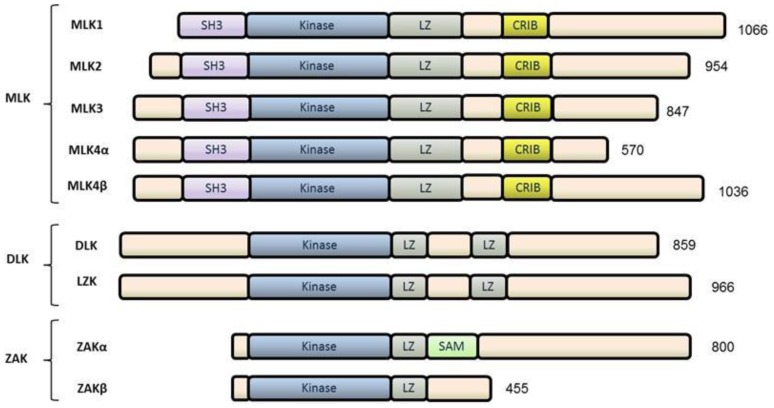
Schematic representation of the mixed-lineage kinase (MLK) family. The MLK family consists of three subfamilies based upon their functional domain arrangements. The MLK subfamily contains an N-terminal Src-homology 3 (SH3) domain followed by a kinase domain, a leucine zipper (LZ) motif, a Cdc42-/Rac1-interactive binding (CRIB) motif, and a proline-rich C-terminal region. The DLK subfamily contains a kinase domain followed by two leucine zipper (LZ) motifs that are separated by a short amino acid spacer, and a proline-rich C-terminal region. The zipper sterile-α-motif kinase (ZAK) subfamily consists of two isoforms: ZAKα contains a kinase domain followed by a LZ motif and a Sterile-α motif (SAM) while the N-terminus though LZ motif of ZAKβ is identical to ZAKα but lacks the SAM and extended C-terminal region.

The MLK family belongs to a large family of MAP3Ks and the name “mixed lineage kinase” arose from the fact that the amino acid sequence of their catalytic domains resembles both tyrosine kinases and serine/threonine kinases. Despite the name, biochemical analyses of MLKs has demonstrated only serine/threonine kinase function [11]. Indeed, this family resides within the “tyrosine kinase-like” branch of the human kinome. Of the seven mammalian MLK family members, all have been shown to function as MAP3Ks in various contexts. Based on functionally conserved domain arrangements and sequence similarity, the MLK family can be clustered into three subfamilies: The MLKs, the dual-leucine-zipper-bearing kinases (DLKs), and the zipper sterile-α-motif kinases (ZAKs) [10] (Figure 1).

### 2.1. MLKs

The mammalian MLK subfamily consists of 4 members: MLK1 (MAP3K9), MLK2 (MAP3K10), MLK3 (MAP3K11), and MLK4, which has two alternatively spliced isoforms known as MLK4α and MLK4β [12]. In *Drosophila*, only one MLK subfamily homolog, named Slipper, exists [10]. All four members were demonstrated to possess MAP3K function *in vitro* [13]. These MLKs share several functional domains including an amino N-terminal Src-homology 3 (SH3) domain, a catalytic domain, leucine/isoleucine zipper (LZ) motifs, and a Cdc42-/Rac1-interactive binding (CRIB) motif [11,14,15]. MLK1-4 share 75% sequence similarity in their kinase domains and 65% in their SH3 domains. However, all members of the MLK subfamily have proline-rich carboxyl C-terminal regions with divergent amino acid sequences and poorly understood functions.

### 2.2. DLKs

The DLK subfamily consists of two members: DLK (MAP3K12) and LZK (MAP3K13). Unlike MLKs, DLKs lack an N-terminal SH3 domain and a centrally-located CRIB motif. Instead, the catalytic domains are followed by two LZ motifs that are separated by a 31-amino acid spacer. Similar to MLKs, the C-termini of DLKs are sequence-divergent proline-rich regions of unknown regulatory function.

### 2.3. ZAK

ZAK is the sole member of the third MLK subfamily. It contains a unique functional domain, the sterile-alpha-motif (SAM), which distinguishes it from the other subfamilies [16]. So far, two splice variants have been identified: ZAKα and ZAKβ. ZAKα contains a kinase domain followed by a short LZ motif and SAM domain, while ZAKβ is identical to ZAKα from the N-terminus to LZ motif but then diverges and lacks a SAM domain.

## 3. MLK3

MLK3, also known as MAP3K11, Src-homology 3 (SH3) domain-containing proline-rich kinase (SPRK), protein-tyrosine kinase 1 (PTK1), and slipper (*Drosophila*), was first identified in 1994 [11,14,15]. This 847-amino acid protein is the only member in the MLK subfamily that has been extensively studied in terms of regulation, functions and implications in human diseases [17,18,19,20,21,22,23,24]. MLK3, as its name MAP3K11 implies, functions as a MAP3K to phosphorylate and activate specific MAP2Ks, which in turn activate specific MAPKs. Three major MAPKs are well described: c-Jun N-terminal kinase (JNK)/Stress-activated protein kinase (SAPK), Extracellular-signaling regulated kinase (ERK), and P38 MAPK. Depending upon context, MLK3 is capable of activating each of these MAPKs [10,25,26]. MLK3 can activate the JNK pathway by phosphorylating and activating MKK4/7 [20,27], or on MKK3/4/6 to activate P38 MAPK [28,29]. MLK3 has been reported to activate ERK in both a kinase-independent and kinase-dependent manner. MLK3 can function as a scaffold protein bridging Rapidly-accelerated fibrosarcoma (Raf)-1 to ERK-specific MAP3K B-Raf. This binding results in Raf-1-dependent B-Raf phosphorylation and activation, which leads to activation of MAPK/ERK kinase 1 and 2 (MEK1/2 also known as MAP2K 1/2 or MKK1/2) and consequently ERK phosphorylation and activation. A recent study has shown that MLK1–4 are enzymatically capable of RAF-independent MEK phosphorylation which, ultimately, can lead to reactivation of ERK signaling in melanoma cells with acquired resistance to a B-Raf inhibitor (Vemurafenib) in B-Raf V600E tumors [30].

Similar to MLK3-mediated ERK activation, MLK3 has also been shown to negatively regulate Ras homolog gene family, member A (RhoA), the guanosine triphosphate phosphohydrolase (GTPase), in both a kinase-dependent and kinase-independent manner. In a kinase-independent mechanism, MLK3 can directly bind to and sequester the RhoA specific guanine exchange factor (GEF), p63RhoGEF thereby preventing its activation by G_αq_, thus suppressing or spatial regulating the activation of RhoA [31]. In breast cancer cells, catalytic activity of MLK3 is required to activate JNK, which in turn phosphorylates paxillin, a focal adhesion (FA) complex scaffold protein, on serine 178. This phosphorylation event of paxillin recruits focal adhesion kinase (FAK), which in turn promotes further phosphorylation of paxillin on tyrosine 31 and 118 [32]. This phosphorylated paxillin is thought to compete with the RhoA-specific GTPase-activating protein (GAP), p190RhoGAP, for binding to Src-homology 2 (SH2) domains of p120RasGAP. Thus, upon phosphorylation, paxillin binds p120RasGAP, releasing p190RhoGAP from p120RasGAP, allowing p190RhoGAP to suppress RhoA activity [33].

How MLK3 signaling results in diverse cellular outcomes is still unclear and requires further investigation. It has been shown that subcellular localization of MLK3 may determine how MLK3 regulates its downstream effectors. While MLK3 localization at the plasma membrane is required for maximal JNK activation (described later in MLK3 regulation), MLK3 distribution at the centrosome and on microtubules appears to regulate microtubule organization during mitosis in a JNK-independent fashion [34]. One possible explanation for various cellular localization and function is the ability of MLK3 to interact with several scaffolding proteins forming localized signalosomes. Hence scaffolding proteins may dictate signaling specificity as well as subcellular localization. At least three members of JNK-interacting proteins (JIPs), JIP-1, -2 and, -3, have been demonstrated to serve as scaffold proteins for a MLK3-MKK7-JNK signaling module [35,36]. JIPs have been recognized as the cargos for a molecular motor kinesin, the motor protein that moves along microtubule filaments [37]. The association of MLK3 with JIPs may provide further explanation for the dynamic cellular distribution of MLK3. Interestingly, the JIP-3 variant, JNK/stress-activated protein kinase-associated protein 1 (JSAP1) is also reported to facilitate JNK activation via an MLK3-independent MEKK1-MKK4-JNK module [38]. Additionally, JIP-2 has been shown to facilitate MLK3-dependent P38 MAPK activation by serving as a docking site for recruitment of MLK3, MKK3, and either P38α or P38δ isoforms. Of note, JIP-4 is the only member of the JIP family that does not form a complex with MLK3 [39]. Additionally, a recent study has identified WD40-repeat protein 62 (WDR62) as a novel MLK3 scaffold protein. Interestingly, although WDR62 is known to recruit JNK 1/2 as well as MKK4/7, the JNK specific MAP2Ks, the recruitment of MLK3 to this complex does not appear to enhance JNK phosphorylation. Rather, the association of MLK3 with this scaffold protein is thought to maintain MLK3 in its inactive state [40] thus, theoretically, preventing MLK3 from phosphorylating and activating its downstream effectors.

Another explanation of how MLK3 might stimulate a wide spectrum of biological responses is its ability to turn on a subset of genes through controlling the transcription factor activation. The activator protein-1 (AP-1) transcription factor is a protein complex comprised of JUN and FOS protein dimers. In some contexts, members of the activating transcription factor (ATF) or Jun dimerization protein (JDP) subfamilies can also substitute as partners within these AP-1 dimers. MLK3 can regulate activation of JNK [29,32,41,42,43,44] and ERK [25,44,45], two major modulators of AP-1 transcription factors [46]. JNK phosphorylates and activates c-Jun, while ERK acts through FOS family members. Of note, a positive feedback loop exists among AP-1 members. For example, increased signaling through MLK3-JNK axis upon induced overexpression of MLK3 in non-tumorigenic mammary epithelial cells results in elevated levels of c-JUN and FRA-1 [43]. Similarly, ERK has been shown to phosphorylate and stabilize FRA-1, which indirectly stabilizes c-JUN through its interaction with FRA-1 [47]. Depending upon transcriptional cofactors, AP-1 activation can result in several biological responses including cell differentiation, cell cycle progression, and invasion [46].

In addition to its regulation of the AP-1 transcription factor, the role of MLK3 as an activator of the NF-kappaB (NF-κB) transcription factor has also been established [48]. MLK3 can directly phosphorylate and activate both IκB kinase α (IKKα) and IκB kinase β (IKKβ). It also can form a complex with two other IκB kinase kinases, MEKK1 and NF-κB-inducing kinase, via association with its leucine zipper region; thus MLK3 is critical for activation of the IκB kinase complex (IKC). In unstimulated T cells, the activity of NF-κB is suppressed via its interaction with an inhibitory protein IκB. Upon T cell stimulation, MLK3 is required for activation of IKC which, in turn, phosphorylates IκB. Phosphorylation of IκB promotes its ubiquitination and proteasome-dependent degradation thereby releasing NF-κB from IκB and allowing NF-κB to translocate to the nucleus where it initiates NF-κB-dependent gene expression [48]. Interestingly, a pan-MLK inhibitor was found to block nuclear translocation of p65, a component of NF-κB complex, in estrogen receptor positive (ER+) breast cancer cells. This suggests a critical role for MLKs in regulating NF-κB signaling in cancer cells [49].

## 4. MLK3 Regulation

### 4.1. MLK3 Contains Intrinsic Autoinhibitory Function

**Figure 2:**
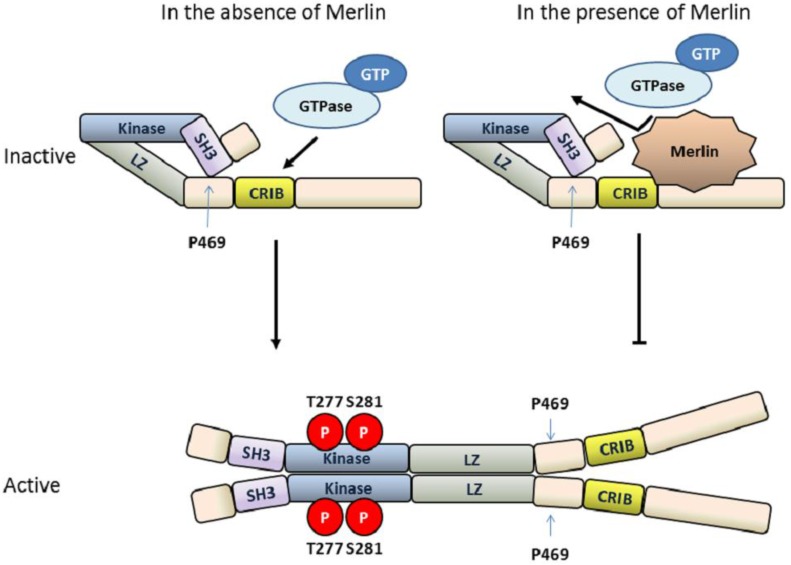
Model for MLK3 autoinhibition, Merlin-dependent inhibition and guanosine triphosphate phosphohydrolase (GTPase)-mediated activation. MLK3 is autoinhibited through intramolecular interactions between its N-terminal Src-homology 3 (SH3) domain and a sequence containing proline 469 that is located between the leucine zipper (LZ) and Cdc42-/Rac1-interactive binding (CRIB) motif. Upon binding to an active (guanine triphosphate (GTP)-bound) Cdc42/Rac1 through the CRIB motif, MLK3 undergoes conformational change, dimerization and autophosphorylation at threonine 277 and serine 281. In the presence of Merlin, direct association between MLK3 and Merlin inhibits MLK3 activation by preventing the activated GTPase from binding to the CRIB motif.

Protein kinases activate their downstream effectors through phosphorylation. To prevent undesired activation, the activities of protein kinases must be tightly controlled. Many protein kinases utilize multiple mechanisms to regulate their activity. MLK3 is no exception. Autoinhibition is commonly used as a repression mechanism for negative regulation of the catalytic activity of protein kinases [50]. MLK3 is one such protein kinase that contains an intrinsic autoinhibitory function that keeps it in its inactive, “closed” conformation, thereby limiting its activity in cells. The intrinsic inhibition of MLK3 is mediated by the N-terminal SH3 domain which serves as an autoinhibitory domain [50]. Indeed, mutation of the conserved tyrosine residue required for SH3-ligand binding to alanine (Y52A) increases MLK3 kinase activity. A typical SH3 domain recognizes and interacts with a tandem PxxP motif on its ligand [51]. However, biochemical assays have shown that MLK3’s autoinhibitory function is mediated through the interaction between its SH3 domain and a region containing a single proline residue at amino acid 469, which is located between LZ domain and CRIB motif, rather within the C-terminal proline-rich region [19]. This critical proline residue is evolutionarily conserved among MLK subfamily members as well as Slipper, MLK3 homolog in Drosophila, suggesting they all utilize this common regulatory mechanism. To release MLK3 from this intrinsic autoinhibition, an additional extrinsic factor is required. Site- directed mutagenesis studies coupled with binding and activity analyses support a model wherein the association of the guanine triphosphate (GTP)-bound cell division control protein 42 (Cdc42) or Ras-related C3 botulinum toxin substrate 1 (Rac1) with MLK3 through its CRIB motif disrupts the SH3-mediated autoinhibition resulting in a conformational change that leads to autophosphorylation and activation of MLK3 [52,53,54] (Figure 2).

### 4.2. Dimerization and Autophosphorylation of MLK3

When MLK3 is in its “open” conformation, MLK3 activity is still tightly controlled by the other mechanisms mediated through additional protein-protein interaction domains. Dimerization of MLK3 is mediated through the LZ motif, supported by the fact that MLK3 mutants lacking the LZ motif fail to form homodimers. Dimerization of MLK3 is a prerequisite for MLK3 autophosphorylation and activation of its downstream c-Jun N-terminal kinase (JNK) pathway [17] (Figure 2). Co-expression of the MLK3 upstream regulator Cdc42 with MLK3 is sufficient to induce homodimerization and autophosphorylation of MLK3 as well as kinase-dependent activation of its downstream signaling effectors. Cdc42 is also required for subcellular targeting of active MLK3 at the plasma membrane leading to maximal activation of the MLK3-MKK7-JNK-c-Jun signaling axis [55]. When LZ-mediated dimerization of MLK3 is destabilized by substitution of a conserved leucine residue with an α-helix-disrupting proline residue, Cdc42 can still induce autophosphorylation of monomeric MLK3 protein. However this monomeric MLK3 is unable to phosphorylate MKK4 on one of its activation specific phosphorylation sites, threonine 258, suggesting that homodimerization of MLK3 is required for proper activation of MAP2K and subsequent activation of the JNK pathway [18].

### 4.3. MLK3 Activity Is Regulated by the Tumor Suppressor Protein Merlin

Tumor suppressor genes encode proteins that are capable of preventing cancer development by inhibiting cell division and promoting cell apoptosis [56]. Merlin is a tumor suppressor protein encoded by Neurofibromatosis type 2 (NF2) gene. Deletion and loss-of-function mutations of NF2 are associated with development and/or invasiveness of several cancers such as schwannomas, meningiomas, ependymomas, mesothelioma, endometrial cancer, ovarian cancer, glioblastoma, and breast cancer [57,58]. In ovarian cancer, Merlin has been shown to negatively regulate MLK3 activity [24,26,59]. Binding of MLK3 to the C-terminus of Merlin inhibits the association of MLK3 with Cdc42 [24] as well as disrupts the MLK3/B-Raf complex [26]. This interaction therefore prevents both kinase-dependent and independent functions of MLK3 and, as a consequence, inhibits activation of MLK3 downstream effectors: B-Raf, JNK and ERK [24,26] (Figure 2). Indeed, knockdown of MLK3 is sufficient to block JNK and ERK activation in a human schwannoma cell line bearing an NF2 loss-of–function mutation and, as a consequence, suppresses proliferation of these cells [26]. Likewise, increased cell proliferation observed upon Merlin silencing in ovarian cancer cells can be reversed by MLK3 knockdown [24].

### 4.4. Heat Shock Protein-Mediated MLK3 Stability

**Figure 3:**
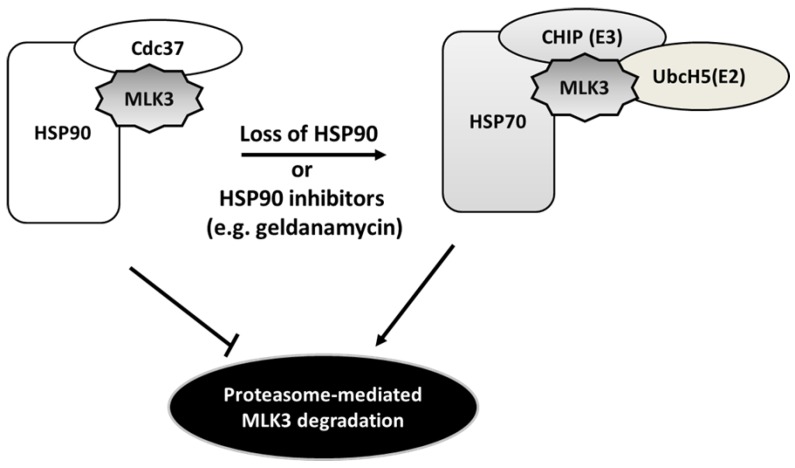
Illustrated model for MLK3 stability. Association of MLK3 with Heat shock protein (HSP) 90 as well as its co-chaperone Cdc37 is required for MLK3 stability. Loss of HSP90 or inhibition of its activity results in dissociation of MLK3 from the HSP90 chaperone complex and promotes interaction of MLK3 with HSP70 along with its co-chaperone E3 ligase carboxyl terminus of Hsc70-interacting protein (CHIP). This association, in turn, recruits E2 ubiquitin-conjugating enzyme UbcH5a, -b, -c and -d, resulting in MLK3 ubiquitination and proteasome-dependent degradation.

Heat shock protein (HSP) 90 is a ubiquitously expressed molecular chaperone that plays pivotal roles in cancer progression [60,61]. HSP90 in concert with its co-chaperone p50^cdc37^ functions to assist protein folding and maturation of a number of oncogenic protein kinases, including Akt, B-RAF, and Src. Thus this chaperone complex is important for the stability and function of several key signaling molecules in cancer [60,62]. In MCF7 breast cancer cells engineered to inducibly express FLAG-tagged MLK3, HSP90 as well as its kinase-specific co-chaperone p50^cdc37^ were identified by liquid chromatography/ tandem mass spectrometry in affinity complexes with MLK3 [20]. Similar to other protein kinase clients, the HSP90/p50^cdc37^ complex interacts with the catalytic domain of MLK3; and is required for MLK3 stability and function. In MCF7 cells, inhibition of HSP90 chaperone activity with geldanamycin, an ansamycin antibiotic, caused downregulation of MLK3 protein levels as well as blocked TNF-induced activation of MLK3 downstream effectors, MKK7, JNK and c-Jun [20]. It was later shown in ovarian cancer cells that HSP90-MLK3 dissociation induced by geldanamycin facilitated association of MLK3 with HSP70 and its co-chaperone E3 ligase carboxyl terminus of Hsc70-interacting protein (CHIP), followed by E2 ubiquitin-conjugating enzyme UbcH5a, -b, -c and -d, resulting in MLK3 ubiquitination and degradation via proteasome pathway [63] (Figure 3). CHIP-mediated degradation of MLK3 is important for suppression of ovarian cancer cell invasion [63]. Of note, HSP90 inhibitors are capable of disrupting the function of various oncogenic kinases and are being examined as potential anticancer therapeutics. Given the findings discussed above, it is possible that the anti-tumor activities of HSP90 inhibitors may result in part from promoting degradation of MLK3.

## 5. MLK3 Transduces Signals from Several Cell-Surface Receptors

**Figure 4:**
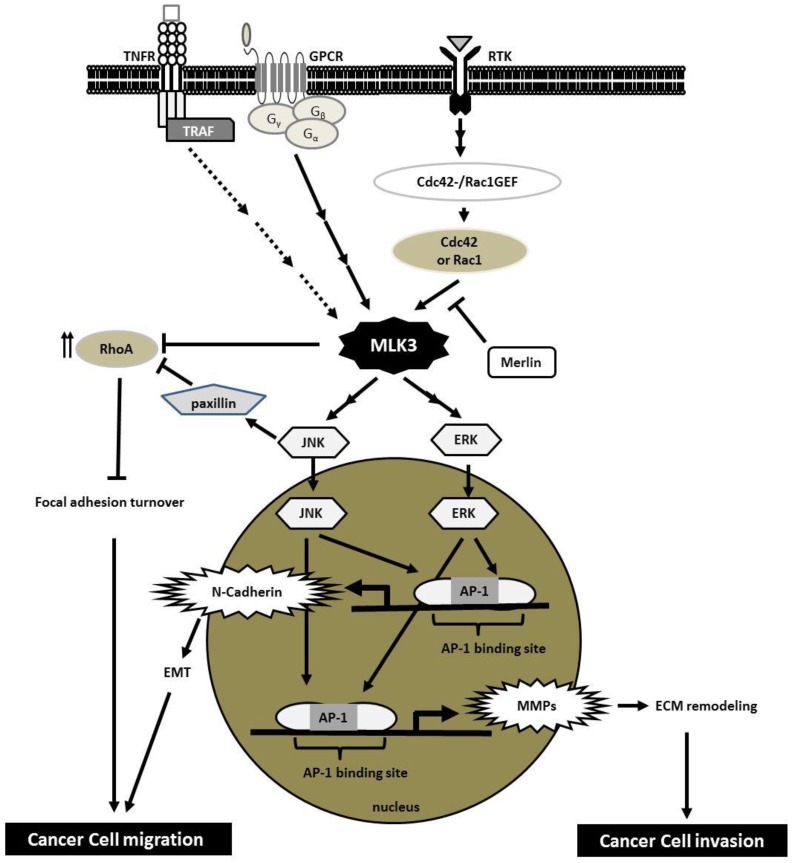
MLK3 signaling in cancer cell migration and invasion. MLK3 transduces signal from multiple cell surface receptors to various downstream signaling effectors to control cancer cell migration and invasion. See text for details.

The ability to transmit signals from various classes of cell surface receptors to multiple signaling cascades makes MLK3 one of the network hubs in cellular signaling [31] (Figure 4). Notably, most cell surface receptors that signal through MLK3 have been shown to play crucial roles in multiple “hallmarks of cancer” including cell proliferation, invasion and cell survival [64]. Likewise, overexpression or mutation of intracellular downstream targets of these receptors commonly occurs in cancer. Indeed, MLK3 has been shown to be overexpressed in breast [43], ovarian [44] and pancreatic [65] cancer. In addition, more than 20% of tumors from patients presenting with microsatellite unstable gastrointestinal cancers harbor the MLK3 P252H mutation, which increases MLK3 activity [66,67].

### 5.1. Receptor Tyrosine Kinases (RTKs)

The RTK family of cell surface receptors have intrinsic tyrosine kinase activity. Upon ligand binding through the extracellular domain, these RTKs undergo dimerization, followed by trans-autophosphorylation within the cytoplasmic domain. Many of these phosphorylated regions serve as docking sites for intracellular proteins ultimately resulting in transduction of extracellular signals to several important downstream cascades including the MAPK pathways. Dysregulation of RTKs, such as members of epidermal growth factor receptor (EGFR) family, or overexpression of their ligands is frequently found in breast cancer [68,69], glioblastomas (GBM) [70,71] and non-small cell lung carcinoma (NSCLC) [72,73], and results in sustained pro-proliferative, pro-invasive and pro-survival signaling [68,69,70,71,72,73,74,75,76,77,78,79,80,81,82].

In various cancer cell lines, MLK3 has been shown to signal through multiple receptors including EGFR [45], hepatocyte growth factor (HGF) receptor, more commonly known as c-MET [32], and Discoidin domain receptor 1 (DDR1) [83]. Depending upon the context, downstream JNK, ERK, or P38 signaling is subsequently activated. Both EGF and HGF can induce several Rac1 or Cdc42 specific Guanine nucleotide exchange factors (GEFs) [84,85,86,87,88]; however, it is still unclear which RacGEF(s) are responsible for MLK3 activation. On the other hand, collagen I-induced DDR1 activation coupling with integrin α2β1 is thought to increase MLK3 activity through a small GTPase Rhoptery associated protein 1 (Rap1) dependent mechanism [83]; however there is no evidence for a direct interaction between MLK3 and Rap1. Depending upon cell types, ligands, and RTKs, MLK3 relays RTK signaling to multiple intracellular targets, resulting in biological responses including cell proliferation, cell migration and invasion, and cell survival.

### 5.2. Chemokine Receptors

Chemokine receptors, a subgroup of cytokine receptors, are a family of seven-transmembrane, G-protein-coupled receptors (GPCRs). GPCR signaling is mediated through association of an intracellular heterotrimeric G-protein with the intracellular portion of the inactive receptor. Upon chemokine activation, GPCRs undergo a conformational change that triggers dissociation of G_α_ from G_β_G_γ_ subunits. These subunits then interact with and transduce signals to multiple pathways including MAPKs [89]. C-X-C chemokine receptor 4 (CXCR4) is a GPCR that is overexpressed in numerous types of malignant cancers, including breast [90], gastric [91], ovarian [92], cervical [93], and colorectal cancers [94]. CXCR4 and its specific ligand, stromal-derived factor 1 (SDF-1), also referred to as CXC ligand 12 (CXCL12), together play a crucial role in cancer survival and metastasis. Notably, CXCL12/CXCR4 appears to be important for site-specific metastasis of several cancers; CXCR4-positive cancer cells invade along CXCL12 gradients thus promoting metastasis to CXCL12-expressing organs such as lymph nodes, lung, livers and bone [95,96].

CXCR4 has been shown to be critical for metastasis of triple negative breast cancer (TNBC). In an experimental model of TNBC, MLK3 has been shown to mediate CXCR4/CXCL12-induced TNBC cell invasion, and knockdown of MLK3 is sufficient to block TNBC metastasis in a xenograft model [32]. However, the mechanism through which CXCR4 activates MLK3 in this context is still unclear. In addition, carbachol, a chemical ligand for the G-protein coupled acetylcholine receptor, can activate MLK3 in lung cancer cells [31].

### 5.3. Tumor Necrosis Factor Receptor (TNFR)

TNFRs, another subgroup of cytokine receptors, are membrane-bound receptors that bind TNF ligands and play key roles in immune homeostasis to promote survival or apoptosis of cancer cells depending on the specific conditions [97,98]. Upon ligand activation, TNFR recruits an adaptor protein, TNFR-associated factor (TRAF) followed by other intracellular signaling proteins.

The consequence of TNFR signaling in cancer is still controversial; depending upon the specific circumstances it can either promote or suppress tumor progression. How TNFR signals to MLK3 in cancer cells is also not known. However it is worth mentioning that TNF-α was the first ligand identified to activate MLK3 [42]. TNF-α stimulates the interaction between MLK3 and TRAF2 and this is crucial for MLK3-JNK signaling [99,100]. Of note, the mechanism of TRAF2-dependent MLK3 activation is still unclear as TRAF2 lacks a kinase domain and, thus, is unable to phosphorylate MLK3. However, two possibilities have been considered: 1) TRAF2 may recruit a protein kinase to phosphorylate and activate MLK3, or 2) the interaction of MLK3 with TRAF2 causes MLK3 to undergo conformational change leading to an increase in its kinase activity [101]. In addition, TRAF6 can promote MLK3 activation [100].

## 6. Implication of MLK3 in Cancer Invasion

### 6.1. Cancer Cell Migration, Focal Adhesion Dynamics and Epithelial-to-Mesenchymal Transition (EMT)

Cell migration is a required step in the process of metastasis, allowing malignant cancer cells to move from a primary tumor to the metastatic sites. The first data to support the role of MLKs in cell migration came from a study in *Drosophila* within which Rac-JNK signaling was shown to be critical for dorsal closure, a process of epithelial cell sheet movement, during *Drosophila* embryogenesis. Of the six *Drosophila* MAP3Ks: MLK, LZK, TGFβ-activated kinase (TAK), apoptosis signal-regulating kinase (ASK), MEK kinase (MEKK) and Tumor progression locus (TPL) homologs, only the MLK homolog, *Slipper*, is critical for JNK-induced dorsal closure in this model [102]. In breast cancer, the requirement of a MLK3-JNK signaling axis in cell migration has also been investigated. Our lab has shown that ectopic expression of MLK3 is sufficient to induce cell migration in both immortalized breast epithelial cells and poorly invasive breast cancer cells, while inhibition of either the MLK3 or JNK pathways, or silencing of MLK3, can block migration of highly migratory, TNBC cells [43].

Cytoskeletal rearrangement and focal adhesion (FA) dynamics are critical for migrating cells and are spatially and temporally regulated by the activities of Cdc42, Rac1 and RhoA GTPases [103]. Cdc42 and Rac1 promote membrane protrusion and regulate the formation of nascent FAs while active RhoA induces the formation of actin stress fibers, promotes maturation of FAs, and stimulates actomyosin contraction, pushing the cell to move forward. In addition, spatial suppression of RhoA activity is necessary for FA turnover [104,105]. Hence the cycling between these RhoGTPases is critical for efficient cell migration. It has been shown that both catalytic activity and scaffolding function of MLK3 are critical for limiting excess RhoA activity and allowing dynamic cell migration. In breast cancer, MLK3 signals through paxillin, which has been shown, in other settings, to activate p190RhoGAP [106]. Depletion of MLK3 or inhibition of its activity results in elevated RhoA activity, excessive FA and stress fiber formation, and decreased cell migration in highly invasive breast cancer cells [32]. In addition, MLK3-mediated suppression of RhoA activity is also required for lung cancer migration [31]. Unlike in breast cancer, the proposed mechanism for suppression of RhoA is through MLK3 binding and sequestration of p63RhoGEF [31]. Of note, antagonism between Rac1 and RhoA activity is well-documented; under conditions of high Rac1, RhoA activity is suppressed and *vice versa*. One possible mediator of Rac1-driven Rho inactivation is through p190RhoGAP [107]. Since Rac1 is an upstream regulator of MLK3, it is possible that MLK3 could be involved in establishing this Rac1-RhoA antagonism by transducing signals from active Rac1 to suppress RhoA via regulation of the activity of 190RhoGAP in JNK-paxillin dependent manner.

Most human cancers are derived from epithelial tissue, the polarized cells that form tight cell-cell contacts and line the cavities and the surface of organs [108]. In cancer cells, the loss of cell polarity and cell-cell adhesion results in EMT, which strongly enhances cancer cell motility and cancer cell invasion. In prostate cancer, MLK3 facilitates the collagen type I-induced EMT switch. In this model, MLK3 transduces signaling from two collagen receptors, α2β1 integrin and discoidin domain receptor 1 (DDR1), leading to a MKK7-JNK-mediated increased expression of EMT marker, N-cadherin. Interestingly, among 12 human MAP3Ks (MEK kinase 1-4, TAK1, ASK 1/2, MLK1-3, DLK and LZK) that were investigated for involvement in promoting EMT phenotype, only MLK3 knockdown was found to be sufficient to block JNK activity, collagen I-induced cell scattering and N-cadherin expression [83].

### 6.2. Cancer Cell Invasion and Metastasis

The ability of cancer cells to promote extracellular matrix (ECM) remodeling and invade through the layer of ECM is a key for their invasiveness and metastatic capability. ECM, a complex network of macromolecules secreted by cells, not only functions as a structural element in tissue but also serves as a stimulus for adjacent cells, leading to several biological responses such as cell migration [109]. Of note, the most abundant type of ECM protein in the human body is collagen [110]. More than 90% of the collagen in vertebrates is fibrillar collagen type I [111], while collagen type IV is a major component of basement membrane, which functions as a barrier separating epithelium from surrounding stroma [112]. The importance of MLK3 in cancer cell invasion has been demonstrated in several types of cancer including breast cancer [32], ovarian cancer [44,63,113], melanoma [114] and non-small cell lung cancer [115]. Though the mechanism of MLK3 in cell invasion is still unclear, inhibition of MLK3 is sufficient to block *in vitro* invasion of cancer cells through Matrigel, which resembles the process of cancer cells breaching through the basement membrane during early steps of cancer metastasis. Blocking ECM invasion could, in theory, result in failure of cancer cells to form distant metastases. Indeed, two research groups have reported that stable knockdown of MLK3 in TNBC cells is sufficient to block formation of pulmonary micrometastases and lymph node metastases [32,116].

One mechanism through which MLK3 may regulate cancer cell invasion is by controlling of expression of matrix metalloproteinases (MMPs). Specific MMPs are required for ECM remodeling that occurs during tissue development and cancer invasion [117]. For example, MMP-1, -2, -7, -8, -13 and MT1-MMP were found to be responsible for type I collagen degradation while collagen type IV, a component of basement membrane, is a substrate of MMP-2, -3, -7, -9, -10, and -13 [118]. Of note, several invasive cancer cells also express high level of MMPs [117,118,119]. In ovarian cancer, the MLK3-ERK-AP1 axis is responsible for production of MMP2 and 9, suggesting that MLK3 may facilitate cancer invasion, in part, through upregulation of MMPs [44,63].

## 7. Other Functions of MLK3

### Cancer Cell Proliferation and Viability

Sustained proliferative signaling is one of the hallmarks of cancer [64] and one key axis that can control such signaling is the Ras/Raf/MEK/ERK [47,120]. In this context, B-Raf, a serine/threonine kinase, acts as a MAP3K relaying Ras signaling to the ERK MAPK pathway [47,120]. In certain cancer models, MLK3 is required for EGF-induced B-Raf activation by providing scaffolding for Raf-1/B-Raf complex; thus this scaffolding function of MLK3 allows Ras-dependent, activated Raf-1 to phosphorylate and activate B-Raf, resulting in increased ERK activity [26,45]. Interestingly, a gain-of-function mutation in B-Raf is frequently found in cancers including melanoma [30] and papillary thyroid carcinoma [121]. Approximately 90% of B-Raf mutations found in melanomas and papillary thyroid carcinomas are point mutations resulting in substitution of of a valine with glutamic acid at amino acid 600 (B-Raf V600E) [30]. This phosphomimetic B-Raf V600E mutation results in constitutively active B-Raf and sustained ERK signaling. Although the B-Raf inhibitor, vemurafinib, has proved efficacious and increases survival in patients with this B Raf mutation, most patients relapse and become resistant to this treatment [122]. Many mechanisms of vemurafenib resistance exist. A recent study has shown that MLK1, -2, -3, and -4 can contribute to acquired vemurafinib resistance in B-Raf V600E tumor model. RNA sequencing reveals upregulation of MLKs in drug-resistant tumor specimens and biochemical assays have also shown that catalytic activity of MLK1, -2, -3, and/or -4 is required for direct phosphorylation of MEK, in a Raf-independent manner, leading to reactivation of ERK signaling and decreased sensitivity to the B-Raf inhibitor [13].

## 8. Conclusions and Future Directions

Cancer invasion is one of the key steps during the metastatic process. Several signalosomes have been shown to govern the invasiveness of the malignant cells. In this review, we have focused on a protein kinase MLK3 and its role in cancer invasion and metastasis. MLK3 is described as a signaling hub mediating multiple extracellular signals into the intracellular environment. In normal cells, MLK3 activity and localization is tightly controlled by both intrinsic and extrinsic mechanisms including autoinhibition, dimerization, and interaction with other signaling proteins and protein scaffolds. Dysregulation of MLK3 activity and/or increased expression of MLK3 is found in numerous types of malignant cancer and substantial studies provide the evidence for the role of MLK3 in cancer invasion. While multiple signaling mechanisms have been identified, numerous *in vitro* and *in vivo* experiments utilizing different cancer models strongly support the critical role of MLK3 in cancer invasion. Thus MLK3 may serve as a potential target for the development of therapeutics against the metastatic disease.

Despite MLK3 being recognized for its role in cancer migration, invasion and metastasis, its upstream regulators or downstream signaling cascades, and how they contribute to cancer metastasis, have not been fully defined. MLK3 can signal to both JNK and ERK. It is noteworthy that both JNK and ERK are major contributors to the activation of the dimeric AP-1 transcription factors. Several invasion genes are controlled by the AP-1 [123]. Thus identifying MLK3-dependent AP-1 target genes that are critical for cancer progression will not only provide a better understanding of how MLK3 signaling controls cancer invasion and metastasis, but also potentially lead to the development of predictive biomarkers which could be used to select cancer patients most likely to respond to treatments that block MLK3 function.
